# A User-Oriented Intelligent Access Selection Algorithm in Heterogeneous Wireless Networks

**DOI:** 10.1155/2020/8828355

**Published:** 2020-11-24

**Authors:** Gen Liang, Xiaoxue Guo, Guoxi Sun, Jingcheng Fang

**Affiliations:** College of Electronic and Information Engineering, Guangdong University of Petrochemical Technology, Maoming 525000, China

## Abstract

A heterogeneous wireless network (HWN) contains many kinds of wireless networks with overlapping areas of signal coverage. One of the research topics on HWNs is how to make users choose the most suitable network. This paper designs a user-oriented intelligent access selection algorithm in HWNs with five modules (input, user preference calculation, candidate network score calculation, output, and learning). Essentially, the input module uses a utility function to calculate the utility value of the judgment parameter; the user preference calculation module calculates the weight of the judgment parameter using the fuzzy analysis hierarchy process (FAHP) approach; the candidate network score calculation module calculates the network score through a fuzzy neural network; the output module calculates the error between the actual output value and the expected output value; and the learning module corrects the parameter of the membership function in the fuzzy neural network structure according to the error. Simulation results show that the algorithm proposed in this paper can enable users to select the most suitable network according to service characteristics and can enable users to obtain higher gains.

## 1. Introduction

In recent years, the continuous development of different wireless network technologies has led to the emergence of various wireless networks, such as the cellular network, the wireless local area network (WLAN), and the wireless metropolitan area network (WMAN). These networks differ in terms of signal coverage, bandwidth, time delay, frequency, etc. [1]. Cellular networks, for example, provide a wide signal coverage and offer excellent mobility support. Meanwhile, WLANs based on the IEEE802.11 standard use 2.4 GHz or 5 GHz frequency bands to communicate, providing users with high-speed data transmission within a limited area. In addition, World Interoperability for Microwave Access (WiMAX) based on the IEEE802.16 standard adopts such technologies as multi-input multi-output (MIMO) and orthogonal frequency division multiplexing (OFDM) and can provide a wide signal coverage and high-speed data transmission rate.

**Figure 1:**
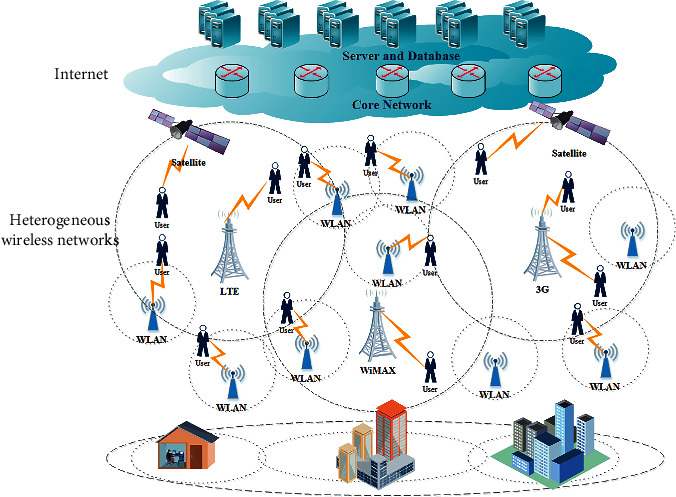
Architecture of HWNs.

By deploying access points of other types of wireless networks, namely, WLAN and WiMAX, heterogeneous systems with multiple networks, as well as overlapping areas of signal coverage, are gradually established. These systems are called heterogeneous wireless networks (HWNs) [2]. In the development process of HWNs, wireless networks have their own architectures and communication protocols. After the access points of these wireless networks are converged, they are connected to a common core network based on IP protocol [3], enabling users connected to these wireless networks to communicate with each other and access the Internet (Figure 1).

In a HWN, mobile user clients are often in the area with overlapping areas of signal coverage as formed by various wireless networks. This requires users to select the optimal network among different candidate networks based on the quality of the wireless channel transmission, the difference in the processing performance of the wireless network and user service requirements, and other reasons. Therefore, access selection is one of the key technologies for HWNs [4, 5].

Traditional HWN access selection algorithms take the received signal strength (RSS) as the judgment parameter for network selection. Mobile users will then select the network with the highest RSS. Although the RSS-based access selection algorithm is low in complexity and easy to implement, it often causes a serious ping-pong effect. To reduce the ping-pong effect, Hanjin et al. [6, 7] propose an improved method to increase lag time based on the highest RSS algorithm; however, this also causes an increase in access delay. In addition, some access selection algorithms use network load as the judgment basis for network selection and connect users to a network with the lowest load in order to achieve load balancing. While these algorithms improve the resource utilization rate of HWNs, they do not consider the quality of service (QoS) requirements of users and potentially connect them to a poor-quality network, thus failing to guarantee the QoS and the experience (QoE) [8, 9].

In a HWN environment, network access selection cannot be only based on one judgment parameter, but requires a comprehensive consideration of multiple judgment parameters (e.g., RSS, bandwidth, network load, delay, jitter, packet loss rate, movement speed, service price, and energy consumption). Due to the difference in network transmission performance and user service types, these multiple parameters enable users to access the most appropriate network [10].

As multiple decision parameters are needed in access selection, some literature uses the theory of multiple attribute decision making (MADM) for designing access selection algorithms [11]. MADM first collects the data of decision parameters to form a multiattribute decision matrix that can be analyzed in a standardized way. Next, it determines the decision weights of decision parameters by an objective or subjective weighting method. Finally, it obtains the ranking of candidate networks. The MADM algorithm includes many branches, such as simple additive weighting (SAW), weighted product method (WPM), analytic hierarchy process (AHP), gray relation analysis (GRA), and technique for order preference by similarity to ideal solution (TOPSIS) [12, 13].

In access selection, different users have different levels of satisfaction with the same parameter value due to their preferences and the diversity of user services. Therefore, some literature uses the utility theory to design network access selection algorithms. The main idea of the access selection algorithms based on the utility theory is to design different utility functions to convert each decision parameter into a utility value [14]. The utility value is a relative index value. Generally speaking, the utility value of a user's most satisfied parameter value is equal to 1, while the utility value of the least satisfied parameter value is equal to 0. The comprehensive utility value of each network is calculated after the utility value of each decision parameter has been obtained, the comprehensive utility values are ranked, and the network with the highest comprehensive utility value is connected. Commonly used utility function types are as follows: linear function, exponential function, logarithmic function, and sigmoid function [15].

As the fuzzy logic theory is suitable for dealing with uncertain and nonlinear problems, expressing knowledge of fuzzy or qualitative analysis and processing natural language with reasoning closely resembling that of a human being, some literature designs access selection algorithms based on the fuzzy logic theory [16, 17]. The main idea of this kind of algorithm is to first fuzzify each decision parameter and generate an input fuzzy set. This then generates an output fuzzy set through fuzzy rules and fuzzy reasoning and finally obtains the accurate scores of candidate networks by defuzzification. The key to the method based on the fuzzy logic theory is to reasonably define fuzzy sets and fuzzy reasoning rules [18].

In addition, some literature uses game theory [19–21], neural networks, the Markov chain [22, 23], and the optimization method [24–26] to design access selection algorithms.

For the access selection issue in a HWN, although many scholars have proposed solutions, most existing access selection algorithms are designed to select a network with the best comprehensive performance for users, ignoring the user's service characteristics and preferences [27, 28]. Therefore, how to design a user-oriented intelligent access selection algorithm from the user's perspective and according to the user's service characteristics and preferences becomes the motivation of this paper.

At present, although other literature designs access selection algorithms by using MADM, utility theory, fuzzy logic, neural networks, and other methods, respectively, no other literature has been found that designs an access selection algorithm combining utility theory, FAHP, fuzzy logic, and neural networks simultaneously. Furthermore, when designing an access selection algorithm, utility value calculation, weight value calculation, and candidate network score calculation of decision parameters under different services are considered at the same time. Therefore, the algorithm proposed in this paper gives a user-oriented intelligent access selection scheme, which can select the most suitable network for users according to their service characteristics and preferences. This is the main contribution and focus of this paper.

The rest of this paper is organized as follows.  Section 2 reviews the related work.  Section 3 provides a detailed description and calculation step of the algorithm framework. In addition, Section 4 configures simulation environment parameters and discusses the experimental results. Furthermore, Section 5 summarizes the article and introduces further research.

## 2. Related Work

At present, some literature uses mathematical models, such as utility theory, fuzzy logic, and neural networks, to design access selection algorithms. Each mathematical model has certain advantages and disadvantages in the design of access selection algorithms [29, 30]. This paper combines the above methods to design the access selection algorithm and mainly analyzes the related literature that combines multiple methods for access selection.

Considering the characteristics of voice application, video application, and best-effort application, Goyal et al. [31] proposed a nonlinear fuzzy optimization model, in which the fuzzy analytic hierarchy process (FAHP) is used to calculate the weight of a decision parameter. Moreover, utility functions are used to calculate the utility values of decision parameters, such as bandwidth, delay, jitter, bit error rate (BER), and cost. Finally, SAW, TOPSIS, and multiplicative exponential weighting (MEW) are used to calculate the scores of candidate networks. While the algorithm proposed in the literature takes into account the service characteristics, it does not consider user preferences for different candidate networks.

Liang and Yu [32] divided user services into different types and calculated the utility value of each network attribute by using utility functions according to the characteristics of different services. Then, the entropy method and the FAHP are used to calculate the objective and subjective weight of network attributes, respectively. In addition, the FAHP is used to calculate the preference value of users to candidate networks. Finally, the multiple attribute decision making method is used to calculate each candidate network score. While this algorithm can reduce the number of handovers between networks, it cannot adjust the scores of candidate networks based on user satisfaction.

Ahuja et al. [17] designed an access selection algorithm by using the RSS, available bit rate, signal-to-noise ratio, throughput, and BER as decision parameters. The algorithm uses utility functions and particle swarm optimization (PSO) to calculate utility values and weights of the decision parameters, respectively, and then uses a fuzzy logic system to calculate the candidate network score. While the algorithm reduces the number of user handovers between networks, it does not take into account the characteristics of different services.

Habbal et al. [33] combined the context-aware concept with the MADM theory and proposed a context-aware multiattribute access selection approach. First, AHP is used to calculate the weight of each decision parameter and then the TOPSIS method is used to select the best network. While the algorithm can solve the problem of abnormal ranking of candidate networks, it does not consider the service characteristics of different users.

Khan et al. [34] designed an access selection algorithm by using decision parameters, such as delay, jitter, BER, packet loss, communication cost, response time, and network load. The algorithm combines fuzzy logic and MADM. First, the algorithm studies the appropriate place where handover is initiated in the wireless signal coverage area, uses a fuzzy system to eliminate the inappropriate candidate networks, and finally selects the optimal network based on the TOPSIS method. While this algorithm reduces the handover delay, it does not consider the characteristics of different services.

In HWNs where WiMAX, LTE, and WLAN coexist, Liang et al. [35] used the RSS, network load, and user rate demand as decision parameters and calculated the score and bandwidth allocation value of each candidate network through a five-layer fuzzy neural network structure. While the algorithm can modify fuzzy rules according to users' preferences and adjust the resource utilization rate of different networks, the increase in the number of candidate networks and decision parameters may lead to a sharp increase in the fuzzy rule base and increase the time delay in access selection.

Calhan and Ceken [36] proposed a handoff decision algorithm based on an artificial neural network, which uses data rate, cost, and RSS as decision parameters. The algorithm first calculates whether it is necessary to hand off to other networks and then selects the best network among all candidate networks. While the algorithm effectively reduces handoff latency, it does not take into account the user's service characteristics and preferences.

At present, although other literature uses MADM, utility theory, fuzzy logic, neural networks, and other methods to design access selection algorithms for HWNs, such literature only selects a network with the best comprehensive performance for users among all candidate networks and does not fully consider various factors, such as network performance, user service requirements, and user preferences; therefore, it is unable to connect users to the most suitable network [4].

This paper designs a network access selection algorithm by combining utility theory, fuzzy hierarchy analysis, fuzzy logic, and neural networks in a HWN, which includes UMTS, LTE, WLAN, and WiMAX, as well as several modules (input, user preference calculation, candidate network score calculation, output, and learning). The main steps of this algorithm are as follows:

First, user services are divided into three types: voice service, video service, and data service. In the input module, bandwidth, delay, jitter, packet loss rate, and price are used as the decision parameters for access selection, and a utility function is designed for each decision parameter according to the characteristics of different types of services, and the utility value of each judgment parameter for different services is calculated by using the utility function.

Second, in the user preference calculation module, FAHP is used to calculate the weight of each decision parameter for different services. Based on this, fuzzy inference rules for different services are generated.

Third, in the candidate network score calculation module, each candidate network is scored by the following three steps: fuzzification, fuzzy reasoning, and defuzzification.

Fourth, the output module calculates the error between the actual output score of a candidate network and the expected score of a user for the network.

Fifth, the learning module corrects the fuzzy and defuzzified membership function parameters in the candidate network score calculation module. This is accomplished according to the error and through supervised learning in order to obtain the final score of a candidate network. Then, the user selects the network with the highest score.

## 3. System Model

### 3.1. Algorithm Framework Design

This paper assumes that a HWN is composed of UMTS, LTE, WLAN, and WiMAX, and mobile user terminals wander randomly within the overlapping areas of signal coverage of these four network signals. In addition, the performance values of all networks can be obtained periodically and any one network can be accessed through the multimode interface using the access selection algorithm. In addition, it is assumed that the user's service types are voice application, video application, and data application, and the user runs any one of the three services.

**Figure 2:**
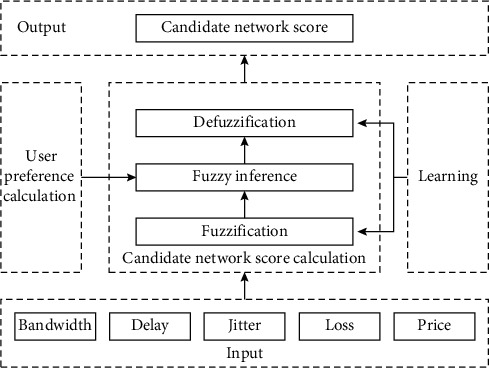
Access selection algorithm framework.

The access selection algorithm framework designed in this paper mainly includes five modules: input, user preference calculation, candidate network score calculation, output, and learning (Figure 2). The main functions of each module are described below:

1. The key role of the input module is to convert the network performance parameter values periodically acquired by users into normalized utility values according to the characteristics of user services and input these values to the candidate network score calculation. In this paper, bandwidth, delay, jitter, packet loss rate, and price are the decision parameters used.
2. The main function of the user preference calculation module is to determine the weight of each decision parameter according to the user service characteristics and then input these weights to the candidate network score calculation. This generates fuzzy inference rules according to the weights.
3. The main function of the candidate network score calculation module is to obtain the score of a candidate network evaluated through three steps, namely, fuzzification, fuzzy reasoning, and defuzzification, according to the bandwidth, time delay, jitter, and other parameters of each candidate network and weight of each parameter and send the score to the output module. This paper uses SCORE to represent the scores of candidate networks and the range of the scores is [0,1].
4. The main function of the output module is to rank the candidate network scores and select the network with the highest score as the final access network. In addition, the module also compares the actual output score of the candidate network with the expected score of the user for the network, calculates the error between them, and transmits the error to the learning module.
5. The main function of the learning module is to adjust the fuzzification and defuzzification parameters in the candidate network score calculation module based on the error between the actual output score of the candidate network and the user's expected network score as to minimize the error.

### 3.2. Input

In access selection, different services have different levels of satisfaction with the same decision parameter value due to different service requirements of users, so the utility function can be used to quantify the satisfaction of users with parameters. The utility function value is a relative index value. Generally speaking, the utility value of a user's most satisfied parameter value is equal to 1, while the utility value of the least satisfied parameter value is equal to 0. The utility function types used in this paper are sigmoid function, exponential function, logarithmic function, linear function, and linear piecewise function, which are defined, respectively, as follows:

Sigmoid function:

(1) $$\begin{array}{c} u\left(x\right)=\frac{{\left(x/a\right)}^{b}}{1+{\left(x/a\right)}^{b}}. \end{array}$$

Exponential function:

(2) $$\begin{array}{c} u\left(x\right)=\frac{e^{cx}-1}{e^{cx}}. \end{array}$$

Logarithm function:

(3) $$\begin{array}{c} u\left(x\right)=d+e{}^{*}\;\ln\left(x+f\right). \end{array}$$

Linear function:

(4) $$\begin{array}{c} u\left(x\right)=gx+h. \end{array}$$

Linear piecewise function:

(5) $$\begin{array}{c} u\left(x\right)=\left\{\begin{array}{cc} 1, & x<i,\\ \frac{j-x}{j-i}, & i\leq x<j,\\ 0, & \text{otherwise}. \end{array}\right. \end{array}$$

The *x* in equations (1)–(5) above is the actual input value of a decision parameter, and *a*, *b*, *c*, *d*, *e*, *f*, *g*, *h*, *i*, and *j* are the curve adjustment parameter values of utility functions. For a beneficial parameter (i.e., bandwidth), the higher the parameter value, the higher the degree of satisfaction, and the utility value is *u*(*x*). For nonbeneficial parameters (e.g., delay, jitter, packet loss ratio, and price), the larger the parameter value, the lower the degree of satisfaction, and the utility value is 1 − *u*(*x*). The actual values of the five decision parameters are inputted into the input module to obtain five utility values between 0 and 1, and these five utility values are transmitted to the candidate network score calculation module.

### 3.3. User Preference Calculation

The analytic hierarchy process (AHP) is one of the commonly used methods for calculating weights, which establishes a comparison matrix by comparing elements in pairs. When the degree of inconsistency of the comparison matrix is not within the allowable range, the comparison matrix needs to be reconstructed.

**Figure 3:**
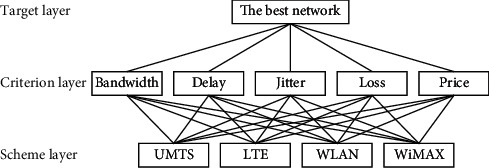
FAHP's hierarchy.

The FAHP is improved on the basis of the analytic hierarchy process. It establishes a consistent pairwise comparison matrix, which has already ensured the consistency of the matrix when it is established [37]. In this paper, the FAHP is used based on the fuzzy consistent matrix to calculate the weights of decision parameters. The main steps are as follows:

 
*Step 1*. Analyze the relationship among decision parameters, candidate network, and target network and construct them into a relationship of three levels (Figure 3). The structure includes a target layer representing the most suitable network, a criterion layer representing decision parameters, and a scheme layer representing each candidate network.
 
*Step 2*. As different decision parameters have different importance to voice service, video service, and data service, according to the FAHP theory, any decision parameter *x*_*i*_ and *x*_*j*_ is compared with each other in terms of degree of their importance and their importance degree ratio *r*_*ij*_ is obtained (Table 1). Then, a fuzzy consistent matrix is constructed using these ratios. According to literature [32], the fuzzy consistent matrices for voice service, video service, and data service are, respectively, shown (Tables 2–4), and the consistency of these matrices is checked according to equation (6). Finally, the weight of each judgment parameter is calculated according to equation (7).

(6) $$\begin{array}{c} \left\{\begin{array}{c} 0\leq r_{ij}\leq 1,\\ r_{ii}=0.5,\\ r_{ij}=1-r_{ji},\\ r_{ij}=r_{ik}-r_{jk}+0.5,\;i,j,k=1,2,\ldots ,n, \end{array}\right. \end{array}$$

(7) $$\begin{array}{c} w_{i}^{sb}=\frac{2}{n\left(n-1\right)}\times \sum_{j=1}^{n}r_{ij}-\frac{1}{n\left(n-1\right)}. \end{array}$$

 
*Step 3*. After the weights of each decision parameter for different services are obtained, the fuzzy inference rules in the candidate network score calculation module are determined according to these weights. In this paper, the number of fuzzy sets is determined for each input decision parameter to 3 (i.e., low, medium, and high), represented by low (L), medium (M), and high (H), respectively. The fuzzy set of the decision parameter *i* in the precondition of fuzzy rules is assumed to be FS_*i*_ and when the fuzzy set FS_*i*_ is L, M, and H respectively, the values of FS_*i*_ are equal to 1, 2, and 3, respectively. Additionally, the fuzzy sets (i.e., low, medium, and high) to which the fuzzy rule conclusion (i.e., score) belongs are determined according to equation (8). Examples of fuzzy inference rules for voice service, video service, and data service are, respectively, shown (Tables 5–7).

(8) $$\begin{array}{c} \text{Score}=\left\{\begin{array}{cc} \text{L}, & 1\leq \sum_{i=1}^{5}{\text{Weight}}_{i}{}^{*}{\text{FS}}_{i}<1.67,\\ \text{M}, & 1.67\leq \sum_{i=1}^{5}{\text{Weight}}_{i}{}^{*}{\text{FS}}_{i}<2.33,\\ \text{H}, & 2.33\leq \sum_{i=1}^{5}{\text{Weight}}_{i}{}^{*}{\text{FS}}_{i}<3. \end{array}\right. \end{array}$$

### 3.4. Candidate Network Score Calculation

**Figure 4:**
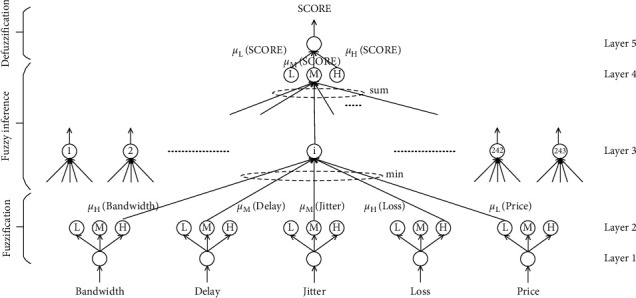
Structure of five-layer neural network.

In Section 3.1, the framework of the access selection algorithm is introduced. In this section, it is designed as a five-layer neural network. Each layer in this network consists of a series of neuron nodes according to the function of each module (Figure 4). The first layer is the input layer. The main function of this layer is to calculate the utility value of the input judgment parameters. The main function of the second layer is for fuzzy processing. The third layer is the fuzzy rules layer, and each node in this layer represents a fuzzy inference rule. The main function of the fourth layer is to perform fuzzy rule inference. The main function of the fifth layer is to perform defuzzification operations to obtain the score of the candidate network.

**Figure 5:**
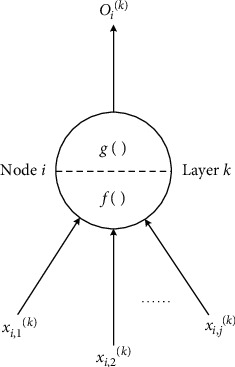
Basic structure of a single neuron.

The specific structure of each neuron node (Figure 4) is shown (Figure 5). According to the basic theory of neural networks, each node contains at least one input data and one output data. Assuming that in Layer *k*, the node *i* contains *j* input data, and all input data are processed by the input data processing function *f*_*i*_^(*k*)^, it can be expressed as *I*_*i*_^(*k*)^=*f*_*i*_^(*k*)^(*x*_*i*,1_^(*k*)^, *x*_*i*,2_^(*k*)^,…, *x*_*i*,*j*_^(*k*)^), and after the input data is processed, it is outputted by an activation function *g*_*i*_^(*k*)^, and the output value is expressed as *O*_*i*_^(*k*)^=*g*_*i*_^(*k*)^(*I*_*i*_^(*k*)^).

The calculation method of each layer (Figure 4) will be described in detail below.

The first layer is the input layer, the main function of which is to calculate the utility values of the input decision parameters according to the service characteristics. Since the proposed algorithm contains five decision parameters (i.e., bandwidth, delay, jitter, packet loss rate, and price), there are five nodes in this layer. The input of nodes is the actual value of each decision parameter. As described in Section 3.2 above, the output of each node in this layer is the utility value, and the value range is [0, 1].

Therefore, the input and output of node *i* in this layer can be expressed as

(9) $$\begin{array}{c} I_{i}^{\left(1\right)}=f_{i}^{\left(1\right)}\left(x_{i,j}^{\left(1\right)}\right)=x_{i,j}^{\left(1\right)},\;i=1,2,\ldots ,5,j=1,\\ O_{i}^{\left(1\right)}=g_{i}^{\left(1\right)}\left(I_{i}^{\left(1\right)}\right)=x_{i,j}^{\left(1\right)},\;i=1,2,\ldots ,5,j=1. \end{array}$$

The main function of Layer 2 is for fuzzy processing. The role of fuzzification is to convert the precise quantities of these inputs into fuzzy quantities and map them into the fuzzy set on the universe of discourse. Since there are five types of data transferred from Layer 1 to Layer 2, three fuzzy sets (i.e., low, medium, and high) are used for each data, so there are 15 nodes in Layer 2. Each node has only one input, which is used to calculate the membership function of each input value belonging to the corresponding fuzzy set. The common membership functions include triangle-shaped membership function, bell-shaped membership function, trapezoidal membership function, and Gaussian membership function. Since Gaussian membership function is easy to derivate and has high efficiency in the learning stage, the Gaussian membership function is adopted as the membership function of this algorithm in this paper, so the input and output of node *i* in Layer 2 can be expressed as follows:

(10) $$\begin{array}{c} I_{i}^{\left(2\right)}=f_{i}^{\left(2\right)}\left(\;\right.x_{i,j}^{\left(2\right)}\left.\;\right)=-\frac{{\left(x_{i,j}^{\left(2\right)}-c_{i}^{\left(2\right)}\right)}^{2}}{{\left(\sigma_{i}^{\left(2\right)}\right)}^{2}},\;i=1,2,\ldots ,15,j=1,\\ O_{i}^{\left(2\right)}=g_{i}^{\left(2\right)}\left(I_{i}^{\left(2\right)}\right)=e^{I_{i}^{\left(2\right)}}=e^{-{\left(x_{i,j}^{\left(2\right)}-c_{i}^{\left(2\right)}\right)}^{2}/{\left(\sigma_{i}^{\left(2\right)}\right)}^{2}},\;i=1,2,\ldots ,15,j=1. \end{array}$$

*c*_*i*_^(2)^ and *σ*_*i*_^(2)^ in the above equation are the mean and the variance of the Gaussian membership function of Layer 2 node *i*, respectively.

Layer 3 is a fuzzy rule layer, and each node in this layer represents a fuzzy inference rule. Since Layer 1 has five input linguistic variables, each input linguistic variable contains three fuzzy sets. In addition, each node in Layer 3 is composed of combinations of fuzzy sets for different input linguistic variables, with a total of 3^5^=243 nodes, and each node corresponds to the precondition of a fuzzy rule. According to the fuzzy logic theory, for all the input data of the same fuzzy rule, the fuzzy AND operation is adopted, and the commonly used fuzzy AND operation is a minimum operation or an algebraic product. This paper uses a minimum operation. Finally, the activation function of each node in this layer only transfers the input function value equivalently, so the input and output of node *i* in Layer 3 can be expressed as

(11) $$\begin{array}{c} I_{i}^{\left(3\right)}=f_{i}^{\left(3\right)}\left(x_{i,j}^{\left(3\right)}\right)=\min\left(x_{i,j}^{\left(3\right)}\right),\;i=1,2,\ldots ,243,j=1,2,\ldots ,5,\\ O_{i}^{\left(3\right)}=g_{i}^{\left(3\right)}\left(I_{i}^{\left(3\right)}\right)=\min\left(x_{i,j}^{\left(3\right)}\right),\;i=1,2,\ldots ,243,j=1,2,\ldots ,5. \end{array}$$

*x*_*i*,*j*_^(3)^ in the above equation represents the *j* input data of node *i* in Layer 3, which is equal to the output data of node *j* in Layer 2 to which node *i* is connected.

The main function of Layer 4 is fuzzy rule reasoning. Among the 243 rules in Layer 3, rules with the same conclusion point to the same node in Layer 4 and perform the fuzzy OR operation on input data pointing to the same node. The most common fuzzy OR operation is the bounded sum or union. This paper uses bounded sum operation. Since there is only one output linguistic variable (i.e., score) in Layer 5, and the linguistic variable contains three fuzzy sets (i.e., low, medium, and high), there are three nodes in Layer 4. The input and output of node *i* in Layer 4 can be expressed as

(12) $$\begin{array}{c} I_{i}^{\left(4\right)}=f_{i}^{\left(4\right)}\left(x_{i,j}^{\left(4\right)}\right)=\underset{j\in C_{i}}{\sum }x_{i,j}^{\left(4\right)},\;i=1,2,3,\\ O_{i}^{\left(4\right)}=g_{i}^{\left(4\right)}\left(I_{i}^{\left(4\right)}\right)=\min\left(1,I_{i}^{\left(4\right)}\right),\;i=1,2,3. \end{array}$$

*C*_*i*_ in the above formula represents the set of nodes in Layer 3 connected with node *i* in Layer 4.

There is only one node in Layer 5, and its main function is to perform the defuzzification operation to obtain the scores of candidate networks. The common defuzzification calculation methods include the mean of maximum method (MOM) and the center of area method (COA). The defuzzification method used in this paper is the COA, so the input and output of the Layer 5 node can be, respectively, expressed as

(13) $$\begin{array}{c} I_{i}^{\left(5\right)}=f_{i}^{\left(5\right)}\left(x_{i,j}^{\left(5\right)}\right)=\sum_{j\in T_{i}}c_{j}^{\left(5\right)}\sigma_{j}^{\left(5\right)}x_{i,j}^{\left(5\right)},\;i=1,\\ O_{i}^{\left(5\right)}=g_{i}^{\left(5\right)}\left(I_{i}^{\left(5\right)}\right)=\frac{I_{i}^{\left(5\right)}}{\sum_{j\in T_{i}}\sigma_{j}^{\left(5\right)}x_{i,j}^{\left(5\right)}}=\frac{\sum_{j\in T_{i}}c_{j}^{\left(5\right)}\sigma_{j}^{\left(5\right)}x_{i,j}^{\left(5\right)}}{\sum_{j\in T_{i}}\sigma_{j}^{\left(5\right)}x_{i,j}^{\left(5\right)}},\;i=1. \end{array}$$

*c*_*j*_^(5)^ and *σ*_*j*_^(5)^ in the above equation represent the mean and the variance of the Gaussian membership function of node *j* in Layer 5, respectively, and *T*_*i*_ in the above equation represents the set of nodes in Layer 4 connected with node *i* in Layer 5.

### 3.5. Output

In Section 3.4, the actual output score of each network is obtained through calculation of the candidate network score. In the output module, the actual output score of the candidate network will be compared with the user's expected score of the network. Then, the errors will be calculated between them and transmitted to the learning module.

Assuming that the actual output score of a candidate network *i* is *y*_*i*_ and the user expected score for the network *i* is *t*_*i*_, then the error between them is defined as

(14) $$\begin{array}{c} e_{i}=t_{i}-y_{i}. \end{array}$$

The main function of the learning module in Section 3.6 is to make the actual output score of a network closer to the expected output score and to minimize the error function based on *e*_*i*_ by adjusting the parameters of the membership function of the fuzzification and defuzzification steps in the candidate network score calculation module. Common error functions include the mean-square error (MSE) and the cross entropy error (CEE). The error function used in this paper is the MSE; namely,

(15) $$\begin{array}{c} E=\frac{1}{2}\sum_{i=1}^{r}{\left(t_{i}-y_{i}\right)}^{2}, \end{array}$$

in which *r* is the number of outputs, expressed as r=1 in this paper. After calculating the error value, the output module transmits it to the learning module to adjust the parameters in the calculation process of candidate networks.

### 3.6. Learning

The main function of the learning module is to minimize the value of equation (15) by adjusting the values of *c*_*i*_^(2)^, *σ*_*i*_^(2)^, *c*_*j*_^(5)^, and *σ*_*j*_^(5)^ of membership functions in the fuzzification and defuzzification steps. In this paper, the gradient descent method is used to solve the adjusted values of *c*_*i*_^(5)^, *σ*_*i*_^(5)^, *c*_*i*_^(2)^, and *σ*_*i*_^(2)^. According to the gradient descent method, assuming the parameter to be adjusted is *ω*, then it is as follows:

(16) $$\begin{array}{c} \Delta \omega =-\eta \frac{\partial E}{\partial \omega }. \end{array}$$

*η* in equation (16) represents the learning rate. In order to accelerate the convergence rate, this paper adopts a variable step learning method based on the mixed momentum term with reference to literature [39] and changes the step size of the learning rate according to the increase of iteration times as shown in the following equations:

(17) $$\begin{array}{c} \omega \left(k+1\right)=\omega \left(k\right)+\eta \left[\left(1-\alpha \right)D\left(k\right)+\alpha \;D\left(k-1\right)\right], \end{array}$$

(18) $$\begin{array}{c} \eta =\mathrm{lg}\left(1+\frac{1}{\beta {}^{*}\text{epoch}}\right). \end{array}$$

In the above equations, *α* is the momentum factor, and 0 ≤ *α* < 1. In this paper, the value of *α* is 0.5, *D*(*k*)=−∂*E*/∂*ω*(*k*) is the negative gradient at time *k*, and *D*(*k* − 1) is the negative gradient at time *k* − 1. *η* is a function of training times epoch, and the value of *β* in this paper is 3.

According to literature [35], the learning rules of the parameters *c*_*j*_^(5)^ and *σ*_*j*_^(5)^ of the Layer 5 membership function, respectively, are

(19) $$\begin{array}{c} \Delta c_{j}^{\left(5\right)}=\eta \left(t_{i}-y_{i}\right)\frac{\sigma_{j}^{\left(5\right)}x_{i,j}^{\left(5\right)}}{\sum_{j\in T_{i}}\sigma_{j}^{\left(5\right)}x_{i,j}^{\left(5\right)}},\\ \Delta \sigma_{j}^{\left(5\right)}=\eta \left(t_{i}-y_{i}\right)\frac{c_{j}^{\left(5\right)}x_{i,j}^{\left(5\right)}\left(\sum_{j\in T_{i}}\sigma_{j}^{\left(5\right)}x_{i,j}^{\left(5\right)}\right)-\left(\sum_{j\in T_{i}}c_{j}^{\left(5\right)}\sigma_{j}^{\left(5\right)}x_{i,j}^{\left(5\right)}\right)x_{i,j}^{\left(5\right)}}{{\left(\sum_{j\in T_{i}}\sigma_{j}^{\left(5\right)}x_{i,j}^{\left(5\right)}\right)}^{2}}. \end{array}$$

In addition, the learning rules of the parameters *c*_*i*_^(2)^ and *σ*_*i*_^(2)^ of the Layer 2 membership function, respectively, are

(20) $$\begin{array}{c} \Delta c_{i}^{\left(2\right)}=-\eta \frac{\partial E}{\partial O_{i}^{\left(2\right)}}e^{I_{i}^{\left(2\right)}}\frac{2\left(x_{i,j}^{\left(2\right)}-c_{i}^{\left(2\right)}\right)}{{\left(\sigma_{i}^{\left(2\right)}\right)}^{2}},\\ \Delta \sigma_{i}^{\left(2\right)}=-\eta \frac{\partial E}{\partial O_{i}^{\left(2\right)}}e^{I_{i}^{\left(2\right)}}\frac{2{\left(x_{i,j}^{\left(2\right)}-c_{i}^{\left(2\right)}\right)}^{2}}{{\left(\sigma_{i}^{\left(2\right)}\right)}^{3}}. \end{array}$$

## 4. Simulation and Result Analysis

### 4.1. Setting of Experimental Parameters

This paper uses MATLAB as the simulation platform. The attribute value settings of candidate networks (Table 8) includes two parts: the first part is the default value (i.e., the value before the parentheses) and the second part is the dynamic value (i.e., the value range within the parentheses, which indicates the lowest value and the highest value of an attribute when it changes dynamically).

As described in Section 3.2 above, equations (1)–(5) are used to calculate the utility values of decision parameters in the input module. In the simulation described herein, the utility functions and parameter values of the functions of decision parameters for different services are set (Table 9).

In Section 3.3, the FAHP is used to calculate the weights of the decision parameters. According to Tables 2–4, in the simulation experiment described in this section, the weight setting of each decision parameter in different services is shown (Table 10). According to these weight values, equation (8) is used to generate fuzzy inference rules.

The experiment in this paper is divided into three parts. The first part is to adjust the parameters of each membership function according to the training data under the static network attribute default value and compare the changes of the membership functions of Layer 2 and Layer 5 before and after learning and the changes of the candidate network scores before and after learning. The second part of the experiment is conducted under dynamic network attribute values (i.e., the numerical range in parentheses in Table 8). In this part of the experiment, the network attribute values will change 1,000 times within the range between their lowest and highest values. Through this experiment, the number of times each candidate network is selected and the number of network handover under different services by the algorithm are evaluated. The third part of the experiment is to compare the proposed algorithm with other algorithms.

### 4.2. Analysis on Experiment Results

#### 4.2.1. Adjustment of Membership Function Parameters

**Figure 6:**
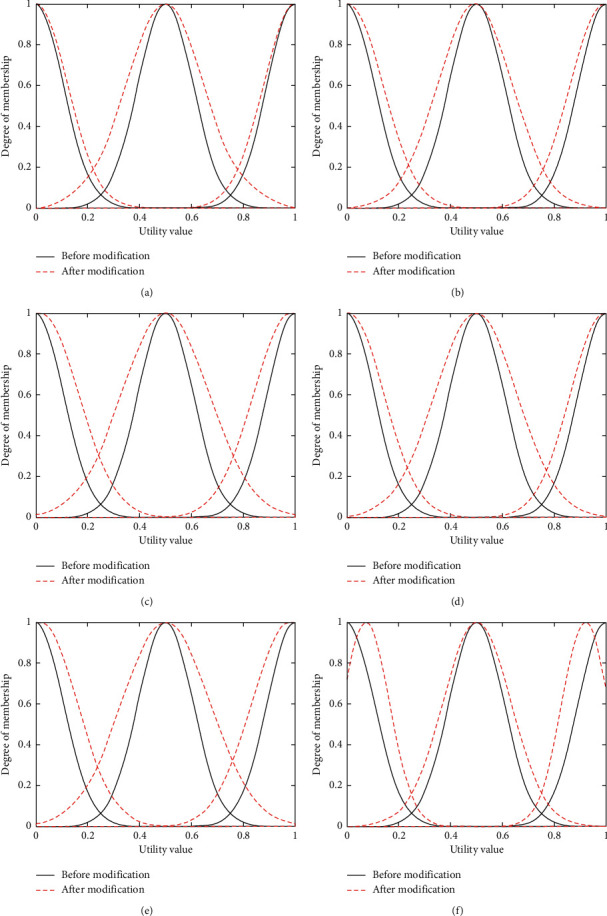
Membership function changes of decision parameters before and after learning under voice service. (a) Change in bandwidth membership function under voice service. (b) Change in delay membership function under voice service. (c) Change in jitter membership function under voice service. (d) Change in loss membership function under voice service. (e) Change in price membership function under voice service. (f) Change in score membership function under voice service.

**Figure 7:**
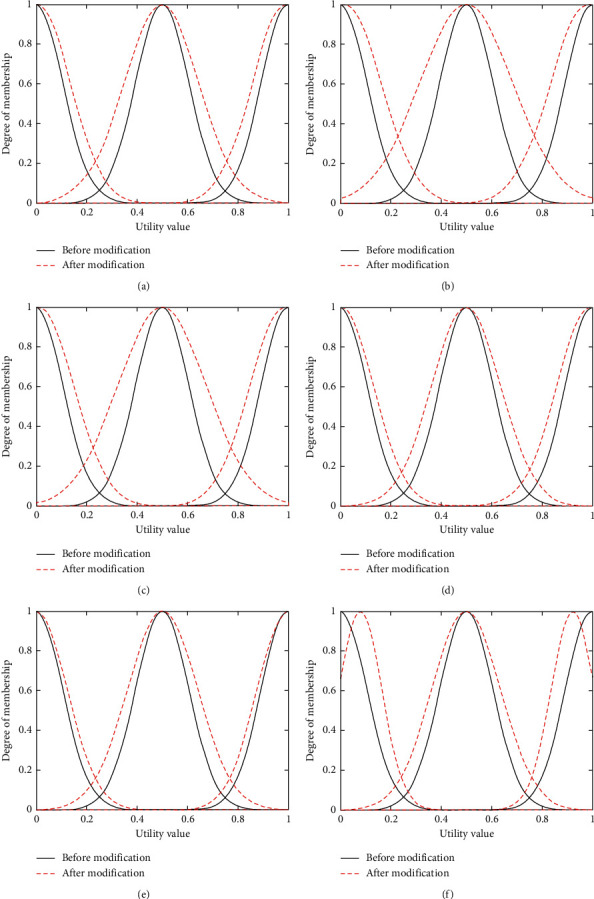
Membership function changes of decision parameters before and after learning under video service. (a) Change in bandwidth membership function under video service. (b) Change in delay membership function under video service. (c) Change in jitter membership function under video service. (d) Change in loss membership function under video service. (e) Change in price membership function under video service. (f) Change in score membership function under video service.

**Figure 8:**
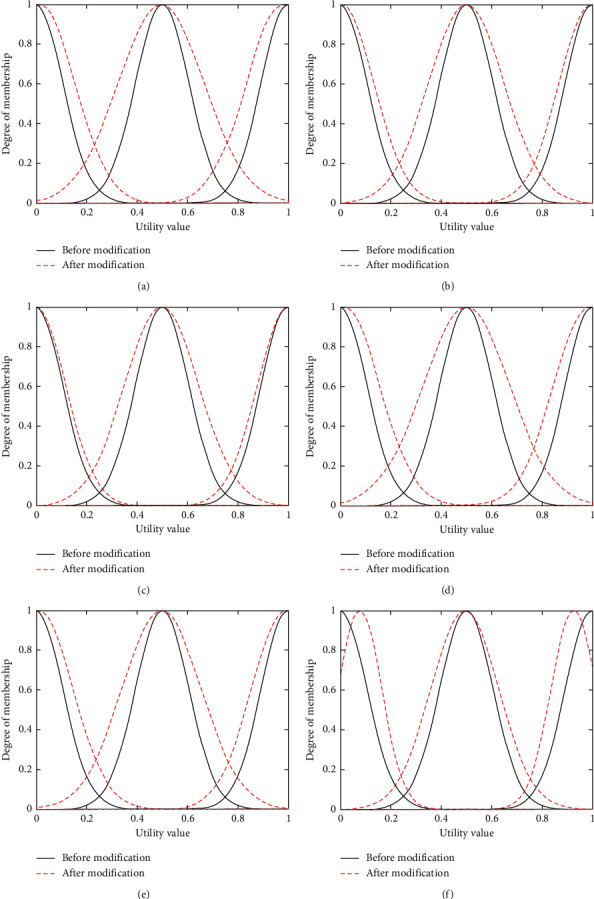
Membership function changes of decision parameters before and after learning under data service. (a) Change in bandwidth membership function under data service. (b) Change in delay membership function under data service. (c) Change in jitter membership function under data service. (d) Change in loss membership function under data service. (e) Change in price membership function under data service. (f) Change in score membership function under data service.

The main purpose of the experiment in this section is to adjust the membership function parameters according to the training samples, so that the candidate network scores calculated after parameter adjustment are closer to the candidate network scores in the training samples. In the experiment described in this section, 5,000 sets of training samples are inputted into the neural network structure shown (Figure 4) under voice service, video service, and data service, and obtain the changes of membership functions under voice service, video service, and data service as shown in Figures 6(a)–6(f), 7(a)–7(f), and 8(a)–8(f) (the black solid lines and red dashed lines in the figures, respectively, represent the curves before and after the adjustment of membership functions).

As can be seen from Figures 6–8, the mean and the variance of membership functions of each input item (i.e., bandwidth, delay, jitter, packet loss rate, and price) and output item (i.e., candidate network scores) have changed. In the learning process, if the mean value becomes greater, it will shift the membership function curve to the right; on the contrary, a lower mean value will shift the membership function curve to the left. The larger the variance, the larger the width of the membership function curve. Conversely, the smaller the variance, the smaller the width of the membership function curve. The changes of the mean and variance parameters change the position and shape of the membership function curve, and the corresponding membership values of low, medium, and high fuzzy sets will also change, thus affecting the score of each candidate network. Taking Figure 6(a) as an example (i.e., the change in bandwidth membership function under voice service), after learning 5,000 sets of training samples, the membership function curve of the decision parameter bandwidth shifts to the right. Under the condition of inputting the same bandwidth value, the membership value of the fuzzy set of low will become larger, so the score of the output candidate network will decrease.

#### 4.2.2. Network Selection under Dynamic Attribute Value

In the previous section, the membership function parameters are adjusted according to the training samples, so that the calculated candidate network scores are closer to the candidate network scores in the training samples. After completing the training, this section will evaluate the average network attribute values of the selected networks under different services, the number of selections of each candidate network, and the number of network handover under 1,000 dynamically changing network attribute values.

**Figure 9:**
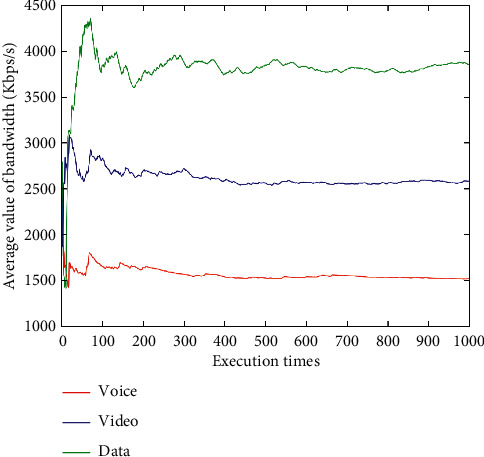
Average bandwidth values of optimal networks under voice, video, and data services.

**Figure 10:**
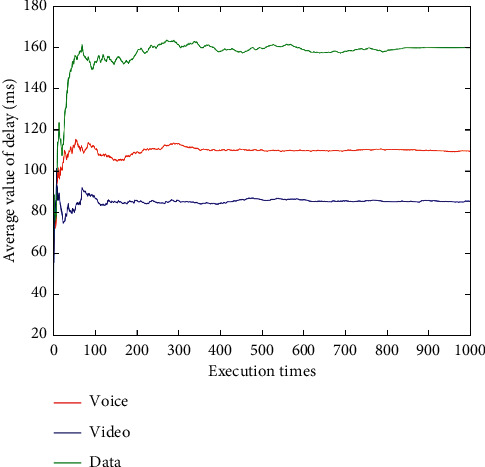
Average delay values of optimal networks under voice, video, and data services.

**Figure 11:**
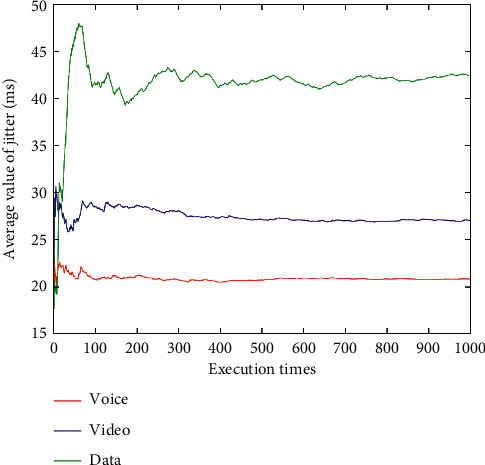
Average jitter values of optimal networks under voice, video, and data services.

**Figure 12:**
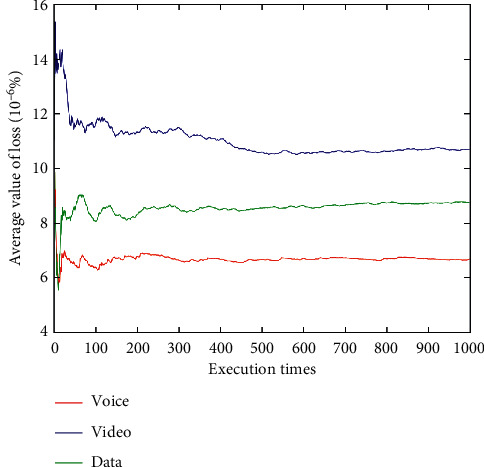
Average packet loss values of optimal networks under voice, video, and data services.

**Figure 13:**
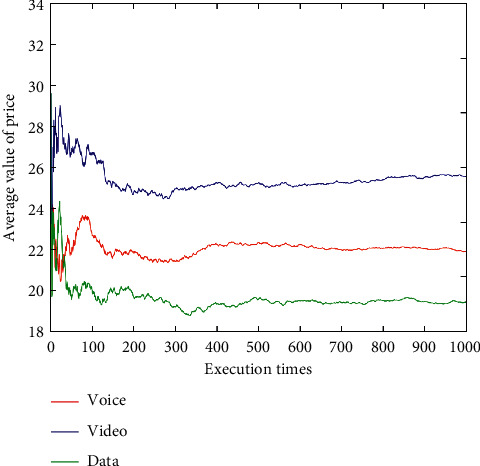
Average price values of optimal networks under voice, video, and data services.

After dynamically changing network attribute values 1,000 times (Figures 9–13), the average network attribute values of the optimal network are selected by voice service, video service, and data service. As can be seen (Figure 9), the average bandwidth value of the data service is the highest due to its higher bandwidth demand, whereas the average bandwidth value of the voice service is the lowest due to its ability to meet its service requirements with a lower bandwidth. As can be seen (Figures 10 and 11), the video service is sensitive to delay, and its average delay value is lower than that of voice service and data service. Meanwhile, the voice service has higher requirements on jitter, and the average packet loss value of the network selected by the voice service is low (Figure 12). In addition, as can be seen (Figure 13 and Table 10), the average price of the network selected by voice service is the lowest because the voice service is heavily weighted by price. Clearly, the algorithm proposed herein can select the most suitable network for users according to the characteristics of each service (Figures 9–13).

**Figure 14:**
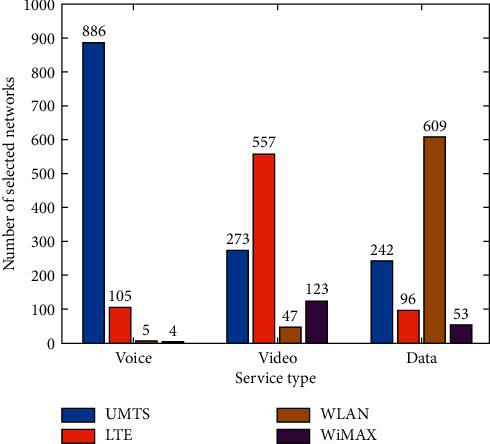
Number of selections of candidate networks under voice, video, and data services.

As can be seen (Figure 14), for the voice service, UMTS and LTE are the networks most selected while WLAN and WiMAX are selected less frequently. LTE is the network most selected for the video service, and UMTS and WiMAX are also selected quite a few times. For the data service, WLAN is the network most selected, while LTE and WiMAX are selected less frequently.

**Figure 15:**
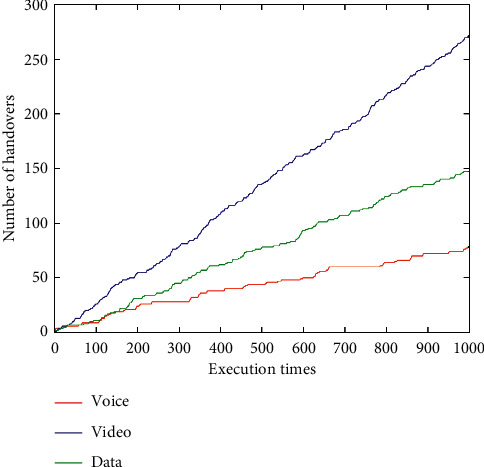
Number of network handovers under voice, video, and data services.

The number of network handovers under different services after 1,000 dynamic changes of network attributes is shown (Figure 15). In this paper, the statistical method of handover times is that if different networks are selected in two consecutive changes of network attribute values, the total number of handovers will increase by 1. As can be seen (Figure 15), since the network most selected by the voice service is UMTS, and WLAN, LTE, and WiMAX are selected less often, the total number of voice service handovers is less than that of video service and data service. For the video service, LTE is the most selected, but other three networks (namely, UMTS, WLAN, and WiMAX) are also averagely selected, resulting in a higher number of handovers. For the data service, WLAN is the most selected; LTE and WiMAX are selected less frequently, so there are a certain number of handovers.

#### 4.2.3. Comparison of Algorithms

To prove the superiority of the algorithm proposed in this paper, this algorithm is compared with three algorithms proposed in other papers (namely, the utility and FAHP algorithm proposed in literature [31], the AHP and TOPSIS algorithm proposed in literature [33], and the fuzzy logic and neural network algorithm proposed in literature [35]). These three algorithms are, respectively, called Algorithm 1, Algorithm 2, and Algorithm 3. For the sake of fairness, the algorithm proposed in this paper and the other three algorithms are all set to be the same for the weights of decision parameters under each service. In addition, in order to compare the influence of the four algorithms on users in access selection, the word “gain” is defined according to literature [11] as shown in the following equation:

(21) $$\begin{array}{c} G_{ij}^{s}=\lambda_{ij}\prod_{k=1}^{n}r_{ik}^{\omega_{k}^{s}}. \end{array}$$

Here, *G*_*ij*_^*s*^ represents the gain obtained for a user whose service type is *s* when accessing network *i* through network *j*. *n* is the number of decision parameters, for *n*=5 in this paper. *r*_*ik*_ is the utility value of the decision parameter *k* in network *i*. *ω*_*k*_^*s*^ represents the weight of the decision parameter *k* when the service type is *s* (i.e., Table 10). In addition, *s* in equation (21) is defined as follows:

(22) $$\begin{array}{c} \lambda_{ij}=\left\{\begin{array}{cc} \alpha , & i=j,\\ \beta , & i\neq j. \end{array}\right. \end{array}$$

In equation (22), when a user selects the same network twice consecutively, let *α*=1, and when the user does not select different networks consecutively, let *β*=0.8.

**Figure 16:**
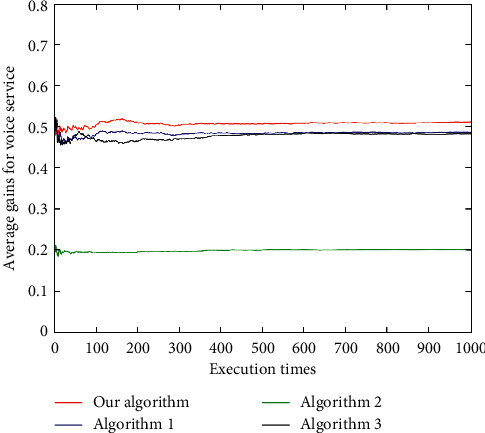
Average user gains under voice service.

**Figure 17:**
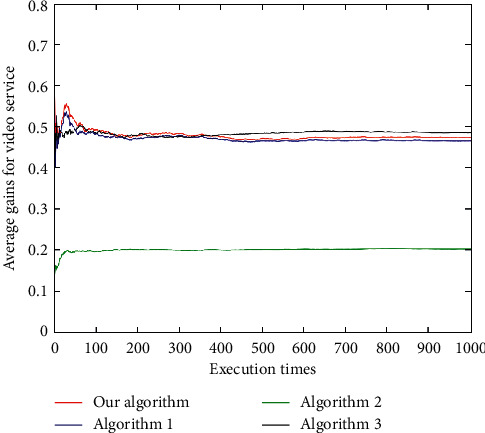
Average user gains under video service.

**Figure 18:**
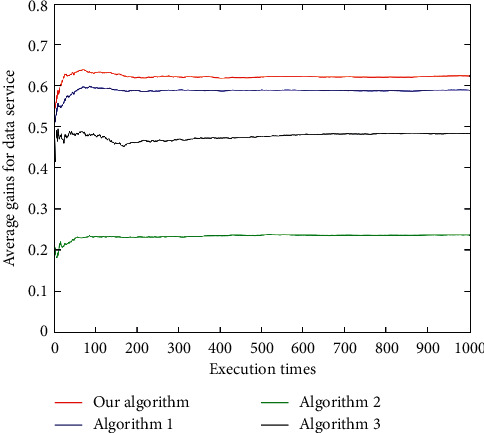
Average user gains under data service.

For the voice service (Figure 16), the average user gain of the algorithm in this paper is better than Algorithm 1 and Algorithm 3, and all are better than Algorithm 2. For the video service (Figure 17), the results of the proposed algorithm and Algorithm 1 and Algorithm 3 are relatively close, and the performance is between Algorithm 1 and Algorithm 3. The results of these three algorithms, however, are not much different. In addition, for the data service (Figure 18), the proposed algorithm has the best average gain, followed by Algorithm 1, Algorithm 3, and Algorithm 2. As can be seen (Figures 16–18), the average gain of the algorithm proposed in this paper is better than that of the other three algorithms due to its dynamically changing network, which enables the algorithm in this paper to select the most suitable network according to the characteristics of user services and preferences.

**Figure 19:**
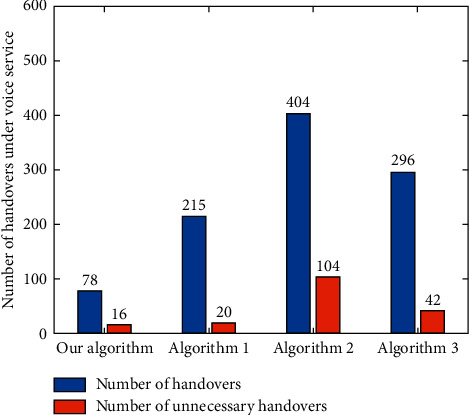
Number of handovers and unnecessary handovers under voice service.

**Figure 20:**
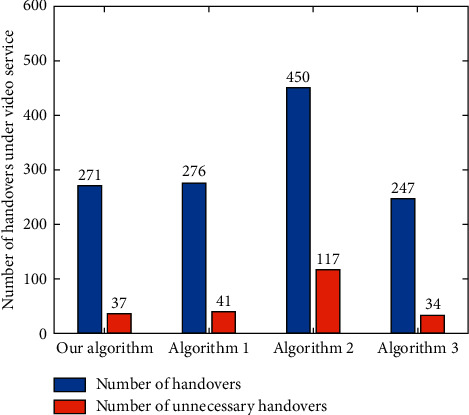
Number of handovers and unnecessary handovers under video service.

**Figure 21:**
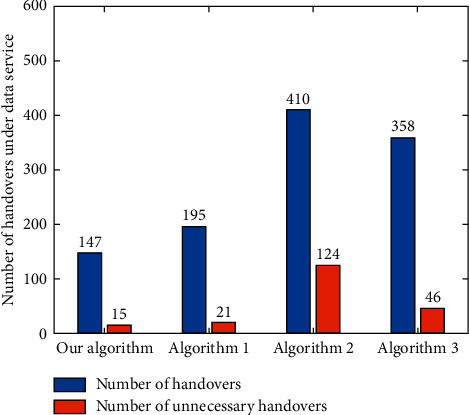
Number of handovers and unnecessary handovers under data service.

According to the definition of “unnecessary handover” given in literature [4], the total numbers of handovers and unnecessary handovers of each algorithm are counted under different services. For the voice service (Figure 19), the numbers of handovers and unnecessary handovers of the algorithm in this paper are less than those of Algorithm 1, Algorithm 2, and Algorithm 3. For the video service (Figure 20), the numbers of handovers and unnecessary handovers of Algorithm 3 are the least, but the performances of the proposed algorithm and Algorithm 1 and Algorithm 3 are not much different. In addition, for data services (Figure 21), the algorithm in this paper has the least number of handovers and unnecessary handovers. Followed by Algorithm 1, Algorithm 3, and Algorithm 2, it can be seen that other algorithms cannot effectively control the potential ping-pong effect under various services, which causes user terminals to switch frequently between different networks. The algorithm proposed in this paper, however, can reduce the number of user handovers between different networks and ensure the QoS and better QoE.

## 5. Conclusions and Outlook

This paper proposes a user-oriented intelligent access selection algorithm in HWNs. The algorithm combines utility theory, fuzzy logic, neural networks, and FAHP; designs modules (input, user preference calculation, candidate network score calculation, output, and learning); and introduces the main functions and calculation steps of each module in detail. The simulation results show that the proposed algorithm enables users to select the most suitable network according to the service characteristics. The next step of this paper is to further consider factors such as handover threshold between networks and user satisfaction, as well as the application of the algorithm in the Internet of things [40], in order to obtain better QoS support and QoE.

## Figures and Tables

**Table 1:** Meaning of importance scale [38].

Scale	Meaning

0.5	Both are equally important
0.6	The former is slightly more important than the latter
0.7	The former is obviously more important than the latter
0.8	The former is strongly more important than the latter
0.9	The former is extremely more important than the latter
0.1, 0.2, 0.3, 0.4	If *x*_*i*_ is compared with *x*_*j*_,*r*_*ij*_ is obtained, and *x*_*j*_ is compared with *x*_*i*_ with results in *r*_*ji*_=1 − *r*_*ij*_
	0.55, 0.65, and 0.75 represent the median value of adjacent grades

**Table 2:** Fuzzy consistent matrix and weights for voice application [32].

Voice	Bandwidth	Delay	Jitter	Loss	Price	Weight

Bandwidth	0.5	0.45	0.15	0.4	0.1	0.1100
Delay	0.55	0.5	0.2	0.45	0.15	0.1350
Jitter	0.85	0.8	0.5	0.75	0.45	0.2850
Loss	0.6	0.55	0.25	0.5	0.2	0.1600
Price	0.9	0.85	0.55	0.8	0.5	0.3100

**Table 3:** Fuzzy consistent matrix and weights for video application [32].

Video	Bandwidth	Delay	Jitter	Loss	Price	Weight

Bandwidth	0.5	0.15	0.4	0.55	0.6	0.1700
Delay	0.85	0.5	0.75	0.9	0.95	0.3450
Jitter	0.6	0.25	0.5	0.65	0.7	0.2200
Loss	0.45	0.1	0.35	0.5	0.55	0.1450
Price	0.4	0.05	0.3	0.45	0.5	0.1200

**Table 4:** Fuzzy consistent matrix and weights for data application [32].

Data	Bandwidth	Delay	Jitter	Loss	Price	Weight

Bandwidth	0.5	0.85	0.95	0.55	0.75	0.3100
Delay	0.15	0.5	0.6	0.2	0.4	0.1350
Jitter	0.05	0.4	0.5	0.1	0.3	0.0850
Loss	0.45	0.8	0.9	0.5	0.7	0.2850
Price	0.25	0.6	0.7	0.3	0.5	0.1850

**Table 5:** Example of fuzzy rules for voice application.

IF					THEN
Bandwidth	Delay	Jitter	Loss	Price	Score

L	L	M	L	H	M
L	L	M	H	H	M
L	M	L	H	L	L
L	H	H	L	L	M
M	L	M	L	M	M
M	L	H	L	L	M
M	M	L	L	H	M
H	L	L	L	L	L
H	L	M	L	L	L
H	H	L	H	H	H

**Table 6:** Example of fuzzy rules for video application.

IF					THEN
Bandwidth	Delay	Jitter	Loss	Price	Score

L	L	M	L	H	L
L	L	M	H	H	M
L	M	L	H	L	L
L	H	H	L	L	M
M	L	M	L	M	L
M	L	H	L	L	L
M	M	L	L	H	M
H	L	L	L	L	L
H	L	M	L	L	L
H	H	L	H	H	H

**Table 7:** Example of fuzzy rules for data application.

IF					THEN
Bandwidth	Delay	Jitter	Loss	Price	Score

L	L	M	L	H	L
L	L	M	H	H	M
L	M	L	H	L	M
L	H	H	L	L	L
M	L	M	L	M	L
M	L	H	L	L	L
M	M	L	L	H	M
H	L	L	L	L	L
H	L	M	L	L	M
H	H	L	H	H	H

**Table 8:** Candidate network attribute value settings.

	Bandwidth (kbps)	Delay (ms)	Jitter (ms)	Loss (%)	Price

UMTS	1100 (700–2000)	60 (30–200)	15 (10–30)	4 (2–10)	20 (5–40)
LTE	2500 (800–4000)	45 (20–150)	20 (15–40)	10 (6–20)	30 (10–45)
WLAN	7200 (1000–8000)	120 (80–300)	60 (30–80)	6 (4–15)	10 (0–35)
WiMAX	4300 (900–6000)	80 (50–250)	30 (20–50)	15 (8–20)	40 (15–50)

**Table 9:** Type of utility function and parameter value setting of function.

	Bandwidth	Delay	Jitter	Loss	Price

Voice	Sigmoid function	Sigmoid function	Logarithm function	Linear function	Linear piecewise function
	*a*=1000	*a*=50	*d*=−2.67	*g*=1/30	*i*=10
	*b*=10	*b*=4	*e*=0.75	*h*=0	*j*=70
			*f*=35		

Video	Sigmoid function	Sigmoid function	Logarithm function	Linear function	Linear piecewise function
	*a*=2500	*a*=100	*d*=−1.35	*g*=1/30,	*i*=15
	*b*=5	*b*=3.5	*e*=0.5	*h*=0	*j*=80
			*f*=15		

Data	Exponential function	Sigmoid function	Linear function	Linear function	Linear piecewise function
	*c*=0.0003	*a*=150	*g*=1/100	*g*=1/30	*i*=20
		*b*=2	*h*=0	*h*=0	*j*=90

**Table 10:** Weight setting of decision parameters in different applications.

	Bandwidth	Delay	Jitter	Loss	Price

Voice	0.1100	0.1350	0.2850	0.1600	0.3100
Video	0.1700	0.3450	0.2200	0.1450	0.1200
Data	0.3100	0.1350	0.0850	0.2850	0.1850

## Data Availability

The datasets generated and/or analysed in this study are available from the corresponding author upon reasonable request.
